# Developmental Decrease of Neuronal Chloride Concentration Is Independent of Trauma in Thalamocortical Brain Slices

**DOI:** 10.1371/journal.pone.0158012

**Published:** 2016-06-23

**Authors:** Joseph Glykys, Kevin J. Staley

**Affiliations:** 1 Department of Neurology, Division of Child Neurology, Massachusetts General Hospital, Boston, Massachusetts, United States of America; 2 Harvard Medical School, Boston, Massachusetts, United States of America; Institut National de la Santé et de la Recherche Médicale (INSERM U901), FRANCE

## Abstract

The intraneuronal chloride concentration ([Cl^-^]_i_) is paramount for determining the polarity of signaling at GABA_A_ synapses in the central nervous system. Sectioning hippocampal brain slices increases [Cl^-^]_i_ in the superficial layers. It is not known whether cutting trauma also increases [Cl^-^]_i_ in the neocortex and thalamus, and whether the effects of trauma change during development. We used Cl^-^ imaging to study the [Cl^-^]_i_ vs. the distance from the cut surface in acute thalamocortical slices from mice at developmental ages ranging from post-natal day 5 (P5) to P20. We demonstrate: 1) [Cl^-^]_i_ is higher in the most superficial areas in both neocortical and thalamic brain slices at all ages tested and, 2) there is a developmental decrease in [Cl^-^]_i_ that is independent of acute trauma caused by brain slicing. We conclude that [Cl^-^]_i_ has a developmental progression during P5-20 in both the neocortex and thalamus. However, in both brain regions and during development the neurons closest to the slicing trauma have an elevated [Cl^-^]_i_.

## Introduction

The action of GABA through GABA_A_ receptors is primarily mediated by the flow of Cl^-^ and to a lesser extent, bicarbonate (HCO_3_^-^) [1]. The driving force for Cl^-^ mediated currents through GABA_A_ receptors depends on the neuronal resting membrane potential (RMP) and the reversal potential of Cl^-^ (based on the local extra- and intracellular [Cl^-^]). If E_Cl_ is significantly more positive than the RMP of a neuron, GABA_A_ receptor activation will depolarize the membrane and lead to excitation or shunting inhibition [2,3]. If E_GABA_ is more negative than RMP, GABA_A_ receptor activation will inhibit the neuron by membrane hyperpolarization.

[Cl^-^]_i_ decreases in a rostral-caudal sequence during development and the neocortex is one of the last regions to develop low [Cl^-^]_i_ [4,5]. However, recent data in the hippocampus illustrated that traumatic sectioning of brain slices increases the [Cl^-^]_i_ in the most superficial neuronal layer [6]. This data raised the possibility that slicing artifacts might have contributed to the observed elevated [Cl^-^]_i_ during early development [7,8]. Furthermore, the relationship between acute brain slice trauma and [Cl^-^]_i_ during early brain development in structures other than the hippocampus has not been addressed.

We previously demonstrated a progressive decrease in [Cl^-^]_i_ in the thalamus and neocortex during early development (post-natal days (P)5-20) using transgenic mice expressing Clomeleon, a genetically encoded fluorophore sensitive to [Cl^-^]_i_, [9]. We found that thalamic neuronal [Cl^-^]_i_ decreased prior to neocortical [Cl^-^]_i_, and that these differences caused region-specific responses to GABAergic anticonvulsants. However, we did not quantify the [Cl^-^]_i_ based on neuron’s depth in the slice at that time. We also did not address the possibility that our results could be explained if the thalamus was simply less susceptible than the neocortex to traumatic increases in [Cl^-^]_i_. Understanding the relation between [Cl^-^]_i_ and the neuron’s depth in an acute slice will better clarify the association between development and [Cl^-^]_i_. It will also give insight on how neurons from different brain regions respond to acute sectioning trauma with regards to the [Cl^-^]_i_.

Here, we re-analyzed our [Cl^-^]_i_ measurements during P5-20 in the ventro-posterior thalamus (VP) and neocortex layer IV/V [9] and correlated it to the neuron’s depth in the slice by developing a method to quantify distance from the irregular contour of the cut surface of the imaged brain slice. We demonstrate that there is a progressive developmental decrease in [Cl^-^]_i_ that is independent of brain slicing trauma in both brain regions, and that the most superficial area of a slice always has an abnormally elevated [Cl^-^]_i_.

## Materials and Methods

### Animals

Postnatal CLM-1 Clomeleon mice (P5–P20; C57bl/6 background) were anesthetized by inhaled isoflurane and decapitated according to a protocol approved by the Institutional Animal Care and Use Committee of the Massachusetts General Hospital Center for Comparative Medicine. The brain was removed and placed in ice-cold aCSF containing (in mM) NaCl (120), KCl (3.3), CaCl_2_ (1.3), MgCl_2_ (2), NaH_2_PO_4_ (1.25), NaHCO_3_ (25), and D-glucose (10) with pH 7.3–7.4 when bubbled with 95% O_2_ and 5% CO_2_. Coronal brain slices, 350–450 μm thick, were cut with a Leica VT1000S Vibratome (Leica Microsystems, Wetzlar, Germany) in aCSF containing 2 mM kynurenic acid [antagonist of ionotropic glutamate receptors to prevent excitotoxic injury (Sigma; St. Louis, MO)]. In the new experiments, brain slices were incubated in 2 mM compared to 0.9 mM MgCl_2_ (as done for the 2009 report) as it is our new protocol aiming to keep slices healthier for longer periods of time. Also, the brain slices were placed in an interface holding chamber at room temperature for 30 minutes and then the temperature was slowly increased and set to 30°C compared to incubating at room temperature only (as done for the 2009 report). Slices were stored for at least 1 hr before being transferred to the recording chamber. After changing the incubation protocol to improve slice health, the baseline [Cl^-^]_i_ decreased in the neocortex from 20.3 ± 20.8 mM (n = 1815 cells) to 14.7 ± 16.2 (n = 258 cells, new experiments) at P10-11, but did not change the Cl^-^ to depth relation as described in the results section.

### Imaging

Two-photon imaging was performed using a Fluoview 1000MPE with prechirp optics and a fast acousto-optical modulator (AOM) mounted on a Olympus BX61WI upright microscope (Olympus Optical) using a 20x water immersion objective (NA 0.95). A mode-locked Ti:Sapphire laser (MaiTai, Spectra-Physics, Fremont, CA) generated two-photon fluorescence with 860 nm excitation. Emitted light was detected through two filters in the range of 460–500 for cyan florescence protein (CFP) and 520–560 for yellow florescence protein (YFP). Two photomultiplier tubes (Hamamatsu Photonics) were used to simultaneously acquire CFP and YFP signals. Slices were perfused with aCSF (0.9 mM MgCl_2_ in the 2009 paper and 2 mM in the new experiments) held at 32–34^o^C and aerated with 95% O_2_−5% CO_2_. Three-dimensional stacks (3D) of raster scans in the XY plane were imaged at *z-*axis intervals of 2 μm.

### Cl^-^ determination

Quantitative measurements on 3D stacks were performed using Image J (National Institutes of Health, freeware) offline. The CFP and YFP z-stack images were loaded and the background level (arithmetic mode of the last slice of a *z*-stack) was subtracted from the entire 3D stack. Next, a median filter was applied to all of the 3D planes. Cells were visually identified, a region of interest (ROI) was drawn around the cell bodies, and the ratio of the YFP/CFP fluorescence intensity was measured. Each cell’s YFP/CFP ratio was converted into [Cl^-^] by the following equation:

$${\left[Cl\right]}_{i}=K\prime_{D}\frac{\left(Rmax-R\right)}{\left(R-Rmin\right)}$$ (1)

where K^’^_*D*_ is the apparent dissociation constant, *Rmax* is the ratio when Clomeleon is not bound by Cl^-^, and *Rmin* when it is completely quenched. The median [Cl^-^]_i_ was used as the distribution of [Cl^-^]_i_ in neurons is skewed to lower values. To avoid excluding occasional neurons whose ratio was below the calibration curve (i.e. when YFP/CFP ratio was greater than *Rmax*), their value was set to 0 mM. The maximum value of Cl^-^ was set to 126.6 mM as this is the value of the extracellular concentration of Cl^-^.

### Cell depth correction

An 8x8 grid (64 squares) was placed on top of the imaged slice using ImageJ. The arithmetic mode (corresponding to the background) of each grid square was calculated for all the *z* planes. Empty space (above the slice) was defined as background equal to 0. The edge of the slice (*ed*), determined in each grid square, was defined when the background increased to >1 in any of the 64 grid squares. Next, the *x*, *y* and *z* position of each neuron (ROI) was obtained. The corrected *z* position of each ROI was obtained by the following equation:

$$Z_{i}=Z_{o}-ed$$ (2)

Where: *Z*_*i*_ is the corrected *z* position of the neuron which gives the distance of the neuron from the cut edge of the slice; *Z*_*o*_ is the original ROI *z* position; *ed* is the *z* position corresponding to the edge of the slice. The computation was done with a macro running in Igor Pro v6.37 (Lake Oswego, OR). Each slice edge position was manually verified.

### Statistics

Histogram bin size was determined by using Scott’s method [10]:

$$BinWidth=3.49\times SD\times N^{-1/3}$$ (3)

Where *SD* is the standard deviation and *N* is the number of observations. As the number of observations and SD varies between experiments and as the imaged *z-*axis interval was 2 μm, we used a single bin size of 6 μm that was obtained by averaging the *BinWidth* from each age group and brain structure (calculated by Eq 3). Deep cells (>150 μm) had low count numbers. Therefore, neurons beyond 150 μm, or if there were less than 20 cells in a bin, were binned together. E_Cl_ was calculated using Nerst equation where [Cl^-^]_o_ was set to 126.6 mM. Mann-Whitney Rank Sum Test (Rank Sum Test) was used to compare non-parametrical data. One-way ANOVA on Ranks with Dunn’s post-hoc test was used to compare multiple groups. Statistical significance was set to *p*<0.05.

## Results

We previously reported a developmental decrease in [Cl^-^]_i_ in the neocortical layer IV/V and ventro-posterior (VP) thalamus at P5-20 measured using transgenic mice expressing Clomeleon [9]. Those [Cl^-^]_i_ measurements were obtained in all Clomeleon-expressing neurons throughout the entire depth of the slice. However, recent data in the acute hippocampal slice demonstrated that the [Cl^-^]_i_ is different at the surface vs. the deeper layers of a slice [6]. Therefore, we went back to the original data to determine if there is a correlation between [Cl^-^]_i_ and cell depth in the acute brain slice, using the same neurons imaged in neocortical and thalamic slices for the 2009 report.

**Fig 1:**
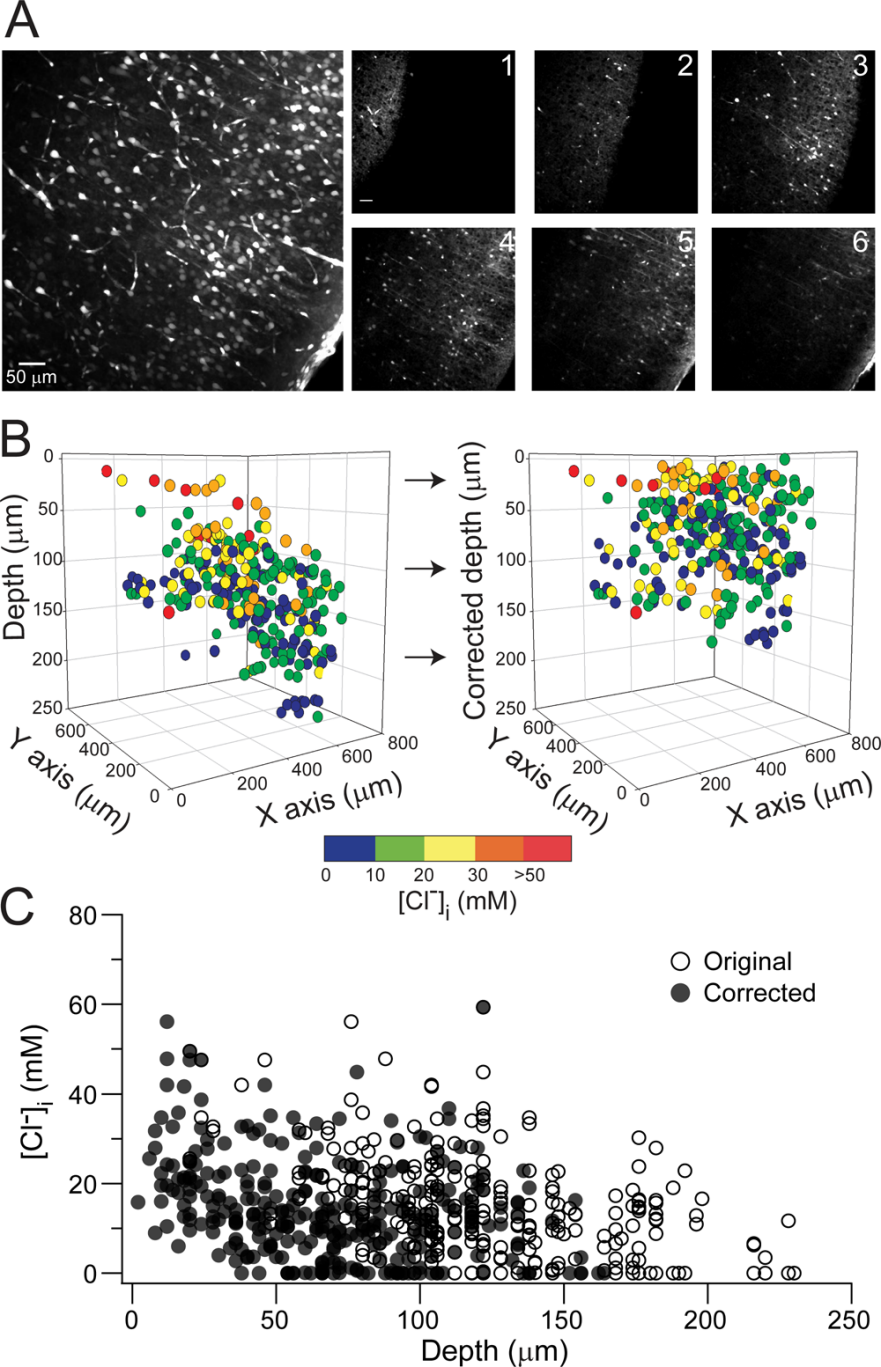
Neuron’s depth correction in an acute brain slice. **(A)** *Left*. Two photon stack image (YFP) of a P10 neocortical slice expressing Clomeleon. *Right panel*, individual z-plane every 20 μm (except #6 which is 15 μm from #5) demonstrating the warp of the imaged slice. **(B)** Pseudo-colored neuronal [Cl^-^]_i_ in 3D space before (*left*) and after depth correction (*right*). Note how the slope of the slice has been corrected. **(C)** [Cl^-^]_i_ against depth of the neuron, original (○) and corrected position (●).

We imaged Clomeleon-expressing neurons in neocortical layers IV/V (frontal to mid-parietal) and VP thalamus at P5–6 (P5), P10–11 (P10), P15–16 (P15), and P20 using multi-photon microscopy. While the slices were imaged, it was evident that the brain slice surface topography was distorted by alterations in the surface tension produced by the imaging objective and the wire harp used to stabilize the slice (Fig 1A). Thus, we first develop a method to correct the position of the neuron based on its relative location to the surface of the slice (Fig 1A and methods). To correct the warping of the slice we segmented each *z* plane into 64 squares and obtained the depth position (*z*-position) of every selected Clomeleon-expressing neuron. Next, we subtracted, from the original *z*-position of each imaged neuron, the location of the edge of the slice in the corresponding grid square. This method allowed us to calculate every neuron’s distance from the cut edge of the slice (Fig 1B and 1C). With the correct depth position of the Clomeleon expressing neurons in the slice we went on to study the relation between [Cl^-^]_i_ and the neuron’s depth at different developmental ages.

### [Cl^-^]_i_ is different between the surface and deep regions in both thalamic and neocortical brain slices at P10

**Fig 2:**
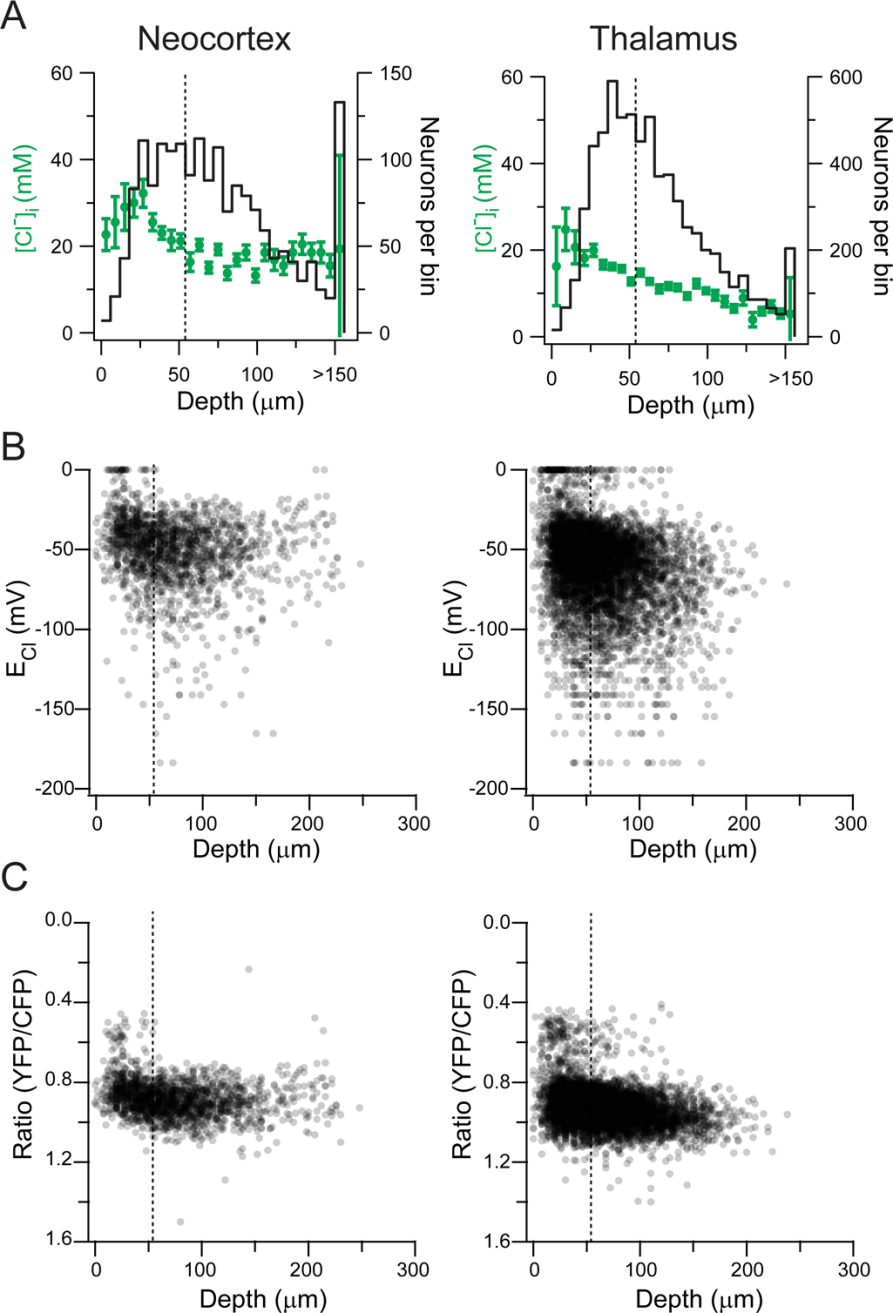
[Cl^-^]_i_ is different between the surface and deep regions in both thalamic and neocortical brain slices at P10. **(A)** [Cl^-^]_i_ distribution against depth (6 μm bins) in the neocortex layer IV/V (*left*) and VP thalamus (*right*) at P10. *Left axis*: [Cl^-^]_I_; *Right axis*: neurons per bin. Error bars represent standard deviation. Dotted line indicates 54 μm of depth. **(B)** E_Cl_ plotted for each neuron against depth in the neocortex layer IV/V (*left*) and VP thalamus (*right*). Dotted line indicates 54 μm of depth. **(C)** Clomeleon YFP/CFP ratio of every neuron depicted in A and B against the neuron’s depth in the neocortex layer IV/V (*left*) and VP thalamus (*right*). Note that the initial 54 μm has a significant amount of neurons with low ratios corresponding with high Cl^-^ values. Each neuron was depicted as a semi-transparent black dot to aid in visualization due to high number of neurons imaged. Dotted line indicates 54 μm of depth.

The neuron’s depth position was binned every 6 μm (see statistics methods). Fig 2A shows the distribution of [Cl^-^]_i_ vs. depth of the neuron in a slice in both neocortex layer IV/V and VP thalamus at P10 and illustrates a strong correlation between [Cl^-^]_i_ and the distance of the neuron from the cut edge of the slice. We defined the superficial layer of the slice as the first 54 μm (9 bin widths) to be able to compare it to the next 54 μm of depth. We analyzed these two depth layers in the slices (0–54 μm and 54–108 μm) because the first 100 μm is where most electrophysiological recordings are done in slice experiments [11–15]. The [Cl^-^]_i_ was significantly higher in the most superficial layer of the slice compared to next 54 μm of depth in the brain slice in both thalamus and neocortex (Neocortex: Superficial (S) [Cl^-^]_i_ 24.8 ± 26.1 mM, *n* = 673; Deep (D) [Cl^-^]_i_ 17 ± 14.5 mM, *n* = 762; Thalamus: (S) 16.5 ± 24.7 mM, *n* = *3029*; (D) 12 ± 15.3 mM, *n* = 2899; Rank Sum Test, *p*<0.001; Fig 2A). There were fewer Clomeleon-expressing neurons in the most superficial layers in both brain structures (Fig 2A), which suggests extensive cell death after sectioning [6]. Further depth subdivisions into more segments did not provide additional useful information. The calculated E_Cl_ had more positive values in the most superficial layers, corresponding to depolarizing actions of GABA_A_R activation at physiological RMPs [16](Fig 2B). Finally, the YFP/ CFP ratios of Clomeleon-expressing neurons (which are independent of its calibration) also demonstrated low values (corresponding to high [Cl^-^]) in the most superficial neurons (Fig 2C). To determine if these initial observations were reproducible in time, new imaging experiments of [Cl^-^]_i_ were performed at P9-11 in layer IV/V neocortical neurons 5 years after the initial data were obtained. The new data also demonstrated a significant difference in [Cl^-^]_i_ between superficial and deep neurons (Rank Sum Test, *p*<0.001).

### Developmental decrease in [Cl^-^]_i_ is independent of traumatic injury

**Fig 3:**
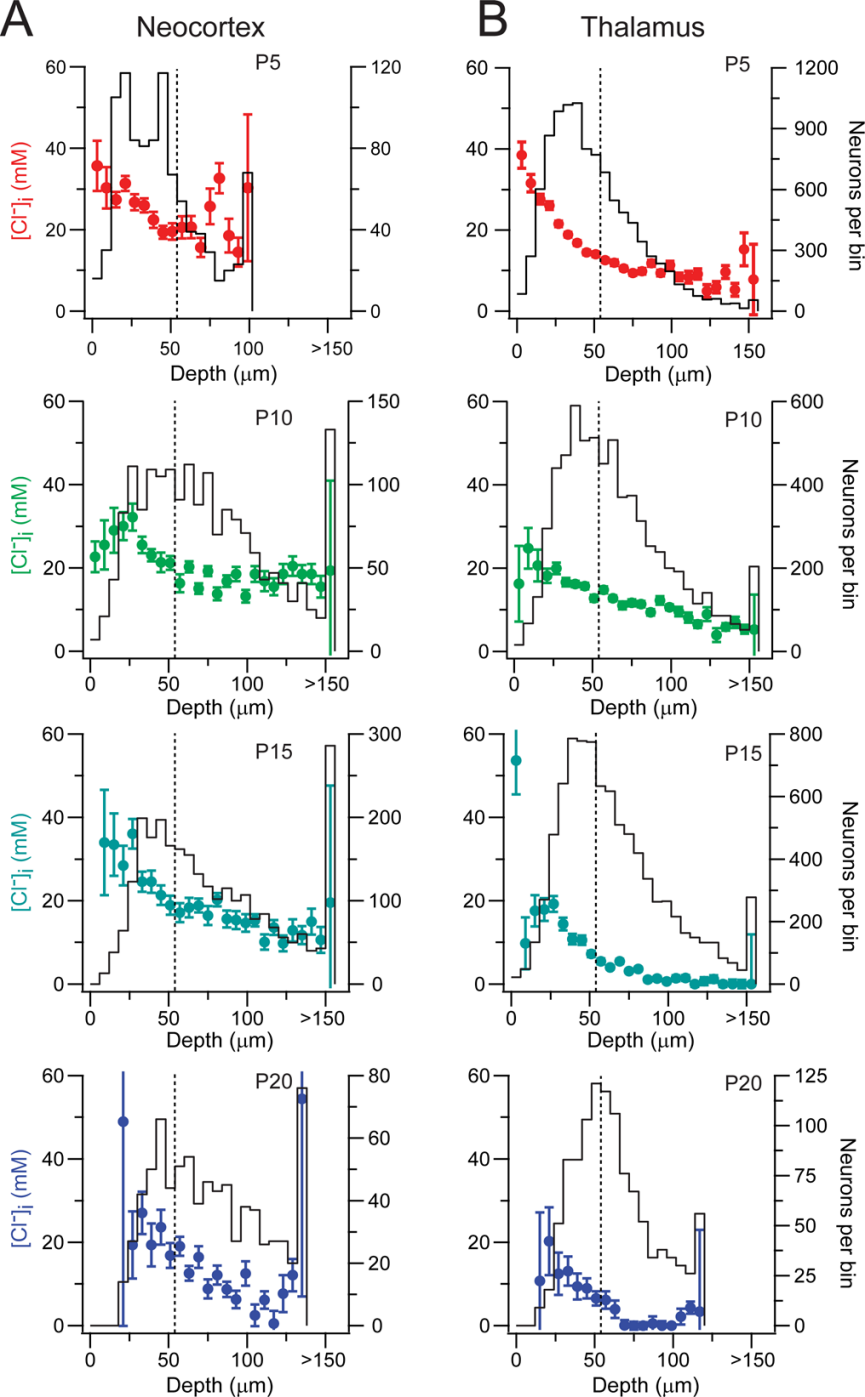
Progressive decrease in [Cl^-^]_i_ in both thalamus and neocortex during early development. **(A)** Neocortex layer IV/V neuronal [Cl^-^]_i_ against depth (6 μm bins) at P5 (*top*), P10 (*middle-top*), P15 (*middle-bottom*) and P20 (*bottom*). *Left axis*: [Cl^-^]_I_; *Right axis*: neurons per bin. Error bars represent standard deviation. Dotted line indicates 54 μm of depth. **(B)** Same as A, but in the VP thalamus at different ages.

**Fig 4:**
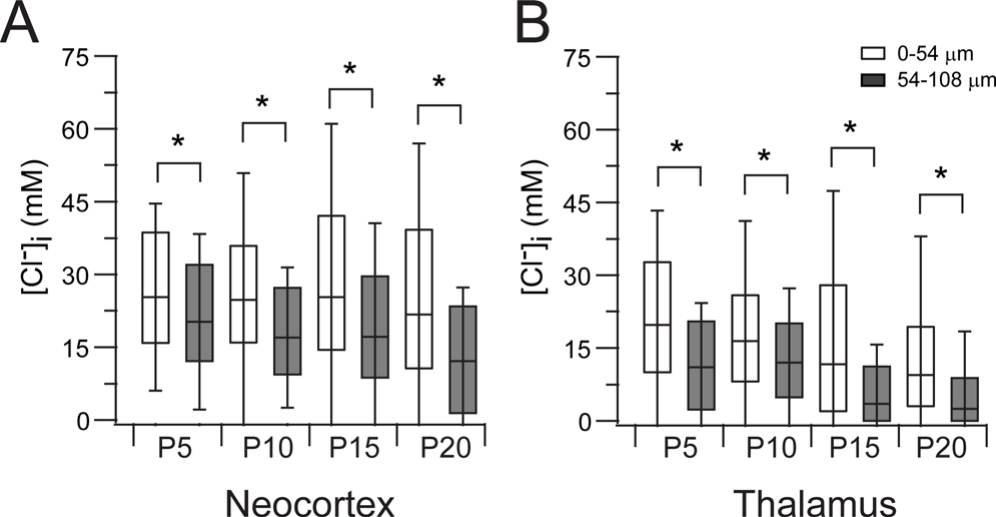
Neurons in the superficial layer of a slice have higher [Cl^-^]_i_. **(A)** Comparison between the neuronal [Cl^-^]_i_ in the superficial layer (□, 0–54 μm) and the next deeper layer (■, 54–108 μm). Box and whisker plot represent the median, 75 and 25 percentile and standard deviation. Note the wider spread of the SD in the superficial layer. **(B)** Same as A but in the thalamus. *, *p*<0.001, Rank-Sum test.

We evaluated if the association between [Cl^-^]_i_ and a neuron’s depth in an acute slice observed at P10 was also present at P5, P15 and P20 (Fig 3). The [Cl^-^]_i_ was higher in the superficial layer of the slice in both thalamus and neocortex at all tested ages (Figs 3 and 4, Rank Sum Test, *p*<0.001). There were fewer Clomeleon expressing neurons in the most superficial layers in both structures, as observed at P10, which accounts for the larger standard deviation (Fig 3 and 4). Interestingly, there is no difference between the [Cl^-^]_i_ of the most superficial regions (<54 μm) in the neocortex at the different ages (One Way ANOVA on Ranks, *p* = 0.09), but there is a difference in the most superficial regions of the thalamus (One Way ANOVA on Ranks *p* = 0.001; post-hoc Dunn’s method *p*<0.05 between all ages, Fig 4). These observations suggest that the thalamic neurons may be more rapidly susceptible to trauma, dying at higher rates during the incubation period prior to Cl^-^ imaging. Also, the proportion of cells in the superficial region is higher in both neocortex (Neo 0.71) and thalamus (Tha 0.63) at P5 and less at the other ages (P10: Neo 0.37, Tha 0.44; P15: Neo 0.36, Tha 0.44; P20: Neo 0.30, Tha 0.42). This suggests that more mature neurons are more susceptible to traumatic death, or that the trauma induced by slicing is more significant in slices from more mature animals.

Despite the difference in [Cl^-^]_i_ between the surface and the deeper areas of an acute brain slice, a progressive developmental decrease in [Cl^-^]_i_ was observed during early maturation in the deeper areas (54–108 μm bin) of the neocortex (One Way ANOVA on Ranks, *p*<0.001; post-hoc Dunn’s method *p*<0.05 between all ages except P15 vs. P10; Fig 4) and thalamus (One Way ANOVA on Ranks, *p*<0.001; post-hoc Dunn’s method *p*<0.05 between all ages, Fig 4), with thalamic [Cl^-^]_i_ decreasing at earlier postnatal ages compared to the neocortex as previously published (Glykys et al., 2009 and Fig 4).

## Discussion

We conclude that neuronal [Cl^-^]_i_ decreases with development throughout the P5-20 interval in both neocortex and thalamus, and that this progressive decrease is independent of slicing trauma. However, in both brain regions, and during development, the most superficial layers close to the edge of the slice have an elevated [Cl^-^]_i_. The same relation between depth and [Cl^-^]_i_ has now been observed in 3 distinct brain regions (neocortex, thalamus and hippocampus) suggesting that the increase in neuronal [Cl^-^]_i_ after traumatic brain injury may be a global phenomenon.

[Cl^-^]_i_ is key in setting the actions of GABA_A_R activation, as Cl^-^ has both a higher permeability and higher extracellular concentration than the other permeant anion, HCO_3_^-^ [1]. A few milimolar change in the [Cl^-^]_i_ can transform the actions of GABA_A_R activation from inhibitory to excitatory as deduced from the Nernst equation, so the developmental decrease in [Cl^-^]_i_ has important consequences for synaptic information transfer. However, data demonstrating differences in [Cl^-^]_i_ and GABA actions between the intact hippocampus versus the acute hippocampal slice has raised questions as to whether the [Cl^-^]_i_ actually decreases during early development [6–8]. Here, we have further analyzed our initial neocortical and thalamic neuronal [Cl^-^]_i_ measurements [9] to take into account the depth of the neurons imaged in the acute slice. We first developed a method to flatten the imaged brain slice that its warped by the surface tension caused in part by the microscope objective and the stabilizing harp. By noninvasively imaging the [Cl^-^]_i_ in hundreds of neurons expressing Clomeleon, we are able to correlate neuron’s depth to [Cl^-^]_i_ as well as the actual YFP/CFP ratio (which is independent of its calibration). Our new analyses demonstrate that the superficial neurons in both the layer IV/V neocortex and VP thalamus had a significantly higher [Cl^-^]_i_ compared to the deeper neurons at all ages. These findings are consistent with our prior results regarding a developmental decrease in [Cl^-^]_i_ in the neocortex and thalamus. They are also consistent with the results of [Cl^-^]_i_ measurements in the neocortex using other techniques [16,17]. Our reanalysis emphasizes the strong relationship between neuronal trauma and [Cl^-^]_i_ [18].

Why is trauma (slice sectioning) associated with neurons having an elevated [Cl^-^]_i_? We hypothesize that this is due to a disruption of the extracellular matrix in addition to the sectioning of neuronal processes. We previously published that degradation of the extracellular matrix by chondroitinase ABC robustly increases the neuronal [Cl^-^]_i_. This and other experiments lead to the idea that the immobile anions set the [Cl^-^]_i_ by displacing Cl^-^ [19]. This paper also argued that chloride co-transporters (including NKCC1 and KCC2) actual role is to facilitate the movement of Cl^-^ and water to maintain the equilibrium conditions established by the local immobile anion concentrations. When the equilibrium changes after damage to the ECM, the chloride co-transporters will alter [Cl^-^]_i_ accordingly. This may explain the beneficial effects of low concentrations of bumetanide (that block NKCC1 activity) after brain injury [6,19–22]. Matrix metalloproteinases, enzymes expressed in neurons and glia that degrade the extracellular matrix, are released during physiological and pathological conditions, including ischemic injury and traumatic brain injury [23–27]. It was recently demonstrated that there is an increase in matrix metalloproteinases protein expression as early as 10 min post injury in the neocortex and peaking at 1 hour in a mouse model of traumatic brain injury [28]. Therefore, we hypothesize that brain slicing, which is a severe type of trauma, leads to physical disruption of the extracellular matrix as well as the release of metalloproteinases from dying and injured neurons as well as from microglia. In addition, increases in neuronal volume accompany the increases in [Cl^-^]_i_ (Glykys et al. 2014), therefore these processes may be relevant to the development of cytotoxic edema after traumatic brain injury.

Interestingly, there are a significant number of Clomeleon expressing neurons in the most superficial layers in both brain structures at P5 yet the proportion of neurons in this superficial layer is lower at P10 an onwards. This suggests that more developed neurons are more susceptible to trauma, but the reasons are beyond the scope of the present study. Possibilities include inability to cope with higher volume and / or [Cl^-^]_i_; neurons with longer/more neurites at older ages; trauma during slicing, for example as a consequence of a physically stiffer brain; higher levels of metalloproteinases; or other traumatic sequela (e.g. elevated [Ca^2+^]). In addition, our results (Fig 3), point out that the first 25 μm of a slice has a lower number of Clomeleon expressing neurons and the highest [Cl^-^]_i_ which progressively decreases with depth. This suggests that the neurons closest to the trauma do not survive and the ones that survive have high [Cl^-^]_i_.

The actions of GABA_A_Rs (excitatory or inhibitory) are not only dependent on the [Cl^-^]_i_ but also dependent on the neuron’s RMP. While we did not measure the RMP in our experiments, other researchers have [16]. As seen in Fig 2, there are a significant number of neurons whose E_GABA_ is more positive than RMP (e.g. –80 mV at P10 as measured by Rheims et al., 2008). The differences in [Cl^-^]_i_ depending on the depth of a neuron in the acute slice should be taken into account when doing electrophysiological measurement of Cl^-^ or cellular physiology.

Measurements of neuronal [Cl^-^]_i_ in the deeper regions in both thalamus and neocortex support the developmental decrease of [Cl^-^]_i_. Further support of the notion of a developmental decrease in [Cl^-^]_i_ comes from the intact preparations of the spinal cord [29,30] and retina, where sectioning trauma is not an issue [31], and the neocortical organotypic culture slice model [32]. In the neocortical organotypic culture slice, there is a progressive decrease in [Cl^-^]_i_ with no depth correlation during different days *in vitro* [32]. In fact, the youngest neocortical organotypic slices have cells with elevated [Cl^-^]_i_ evenly distributed throughout the whole slice. The data from the neocortical organotypic slices in association with our current results suggest that if trauma is taken out of the equation, there is still a developmental decrease in [Cl^-^]_i_. While we have not been able to grow thalamic organotypic slices, based on current results, we expect that [Cl^-^]_i_ in that preparation will be related to the neocortical organotypic slice findings in the same manner that thalamic [Cl^-^]_i_ is related to neocortical [Cl^-^]_i_ in acute slices.

Our results are consistent with our prior study in the hippocampus that show elevated [Cl^-^]_i_ in the superficial layers of the acute hippocampal slice [6]. While the intact hippocampus demonstrates lower [Cl^-^]_i,_ between P5-P7 compared to the acute slice preparation, GABA also has a depolarizing effect in the hippocampal intact preparation at P1-3 [33]. Moreover, there is indirect data demonstrating depolarizing actions of GABA in the neonatal neocortex *in vivo* by using electrophysiological and Ca^2+^ imaging [34]. *In vivo* data regarding neuronal [Cl^-^]_i_ will be key in further assessing the developmental profile of [Cl^-^]_i_ in the hippocampus as well as in the neocortex_._

Future research will clarify the proportion of neurons in which GABA_A_ receptor activation is excitatory, and whether the effects of trauma and development are class dependent (pyramidal neurons, layer specific neurons, subpopulations of interneurons). A handful of neurons with elevated [Cl^-^] may be enough to induce pathological hyperexcitability or synchrony. Finally, the modulation of [Cl^-^]_i_ has clinical implications because a decrease in [Cl^-^]_i_ by a few milimolar, either by development or drugs, improves the efficacy of GABA_A_R positive allosteric modulators such as phenobarbital and benzodiazepines that are frequently used to treat neonatal seizures [9,32,35].
